# Feasibility and safety of a novel 3D-printed biodegradable biliary stent in an in vivo porcine model: a preliminary study

**DOI:** 10.1038/s41598-022-19317-y

**Published:** 2022-09-23

**Authors:** Jae Hyun Kim, Dong-Heon Ha, Eui Soo Han, YoungRok Choi, Jiwon Koh, Ijin Joo, Jung Hoon Kim, Dong-Woo Cho, Joon Koo Han

**Affiliations:** 1 Department of Radiology, Seoul National University Hospital, 28, Yongon-dong, Chongno-gu, Seoul, 110-744 Republic of Korea; 2 Department of Mechanical Engineering, Pohang University of Science and Technology, Pohang, Republic of Korea; 3 Department of Surgery, Seoul National University Hospital, Seoul, Republic of Korea; 4 Department of Pathology, Seoul National University Hospital, Seoul, Republic of Korea

**Keywords:** Biliary tract disease, Preclinical research, Biomedical materials, Bile ducts

## Abstract

To assess the feasibility and safety of a novel 3D-printed biodegradable biliary stent using polycaprolactone (PCL) in an in vivo porcine model. In this animal study using domestic pigs, biodegradable radiopaque biliary stents made of polycaprolactone (PCL) and barium sulfate were produced using 3D printing and surgically inserted into the common bile duct (CBD) of pigs (stent group, n = 12). Another five pigs were allocated to the control group that only underwent resection and anastomosis of the CBD without stent insertion. To check the position and status of the stents and stent-related complications, follow-up computed tomography (CT) was performed every month. The pigs were sacrificed 1 or 3 months after surgery, and their excised CBD specimens were examined at both the macroscopic and microscopic levels. Three pigs (one in the stent group and two in the control group) died within one day after surgery and were excluded from further analysis; the remaining 11 in the stent group and 3 in the control group survived the scheduled follow-up period (1 month, 5 and 1; and 3 months, 6 and 2 in stent and control groups, respectively). In all pigs, no clinical symptoms or radiologic evidence of biliary complications was observed. In the stent group (n = 11), stent migration (n = 1 at 3 months; n = 2 at 1 month) and stent fracture (n = 3 at 2 months) were detected on CT scans. Macroscopic evaluation of the stent indicated no significant change at 1 month (n = 3) or fragmentation with discoloration at 3 months (n = 5). On microscopic examination of CBD specimens, the tissue inflammation score was significantly higher in the stent group than in the control group (mean ± standard deviation (SD), 5.63 ± 2.07 vs. 2.00 ± 1.73; P = 0.039) and thickness of fibrosis of the CBD wall was significantly higher than that of the control group (0.46 ± 0.12 mm vs. 0.21 ± 0.05 mm; P = 0.012). Despite mild bile duct inflammation and fibrosis, 3D-printed biodegradable biliary stents showed good feasibility and safety in porcine bile ducts, suggesting their potential for use in the prevention of postoperative biliary strictures.

## Introduction

Benign biliary strictures can be caused by a wide array of non-neoplastic factors, such as postoperative complications and inflammatory conditions. Iatrogenic causes such as cholecystectomy and liver transplantation (LT) are the most common causes and account for up to 80% of all benign biliary strictures^1,2^. In addition, the incidence of biliary anastomotic stricture, which is a common source of morbidity and mortality, is known to be higher in living-donor LT (LDLT) than deceased-donor LT (19–40% vs. 15–20%)^3–5^. The most important aspect of biliary stricture management is achieving permanent patency and minimizing the need for repeated interventions or surgical treatments. There are several available nonsurgical methods to manage biliary strictures, including large-bore catheterization, balloon dilatation, and stent placement via endoscopic or percutaneous approaches^6–8^. Although these methods are effective in treating biliary strictures, repetitive intervention sessions for catheter exchange or stent removal have a negative effect on patient compliance and quality of life.

Recently, biodegradable stents have been proposed for the treatment of benign biliary stricture^9–11^. Impressively, because biodegradable stents degrade spontaneously, no further interventions are needed, and patients can be discharged without an external biliary drainage catheter. The biodegradable stents used in previous studies were made of polydioxanone (PDO), which is often used to produce surgical sutures. However, according to a previous study^12^, PDO showed a rapid decrease in the mechanical strength with a greater than 50% decrease from the initial value within 14 days. For biliary stents, the maintenance of bile duct patency is crucial; considering that approximately two-thirds of biliary complications occur within 3 months after LT^13^, PDO might not be suitable for biliary stricture management. In contrast, polycaprolactone (PCL), which is a widely used biodegradable material in medical devices, maintains its mechanical strength after 60 days^12^. Chang et al.^14^ demonstrated that PCL maintains its weight after 70 days of degradation in human bile juice.

In addition, rapidly evolving 3D printing technology enables the manufacture of personalized medical devices^15^. Regarding biliary stricture, if it is possible to create a biliary stent after evaluation of the patient’s bile duct anatomy using computed tomography (CT) or magnetic resonance imaging (MRI), biliary strictures could be managed more effectively. In a previous study^16^, we established a rotating rod combined with a 3D printing system and successfully fabricated biodegradable stents with PCL for femoral-iliac artery treatment.

In this study, we assessed the feasibility and safety of a novel 3D-printed biodegradable biliary stent using PCL in an in vivo porcine model.

## Materials and methods

### Ethical statement

This study was approved by the Institutional Animal Care and Use Committee of Seoul National University Hospital (IACUC; No. 20-0096-S1A2) and was performed in accordance with the Guide from our IACUC and the National Institute of Health Guide for the Care and Use of Laboratory Animals. All animals were maintained in a facility accredited by AAALAC International (#001169) in accordance with the Guide for the Care and Use of Laboratory Animals, 8th edition, NRC (2010). All methods are reported in accordance with ARRIVE guidelines.

### Development of radiopaque biodegradable biliary stent using rotating rod-combined 3D printing system

PCL (*M*_W_ = 43,000–50,000, PolyScience Inc., Warrington, PA, USA) was used as a biodegradable material. PCL is a bioresorbable polymer that has been used in FDA-approved medical devices, such as implants, drug delivery devices, and sutures. For radiopacity of the stent, a fine powder of barium sulfate (E-Z-HD; E-Z-EM Canada Inc., Quebec, Canada) was used. Barium powder was agitated uniformly in molten PCL on a hot plate (PCL with 25% [w/w] BaSO_4_, 110 °C).

**Figure 1:**
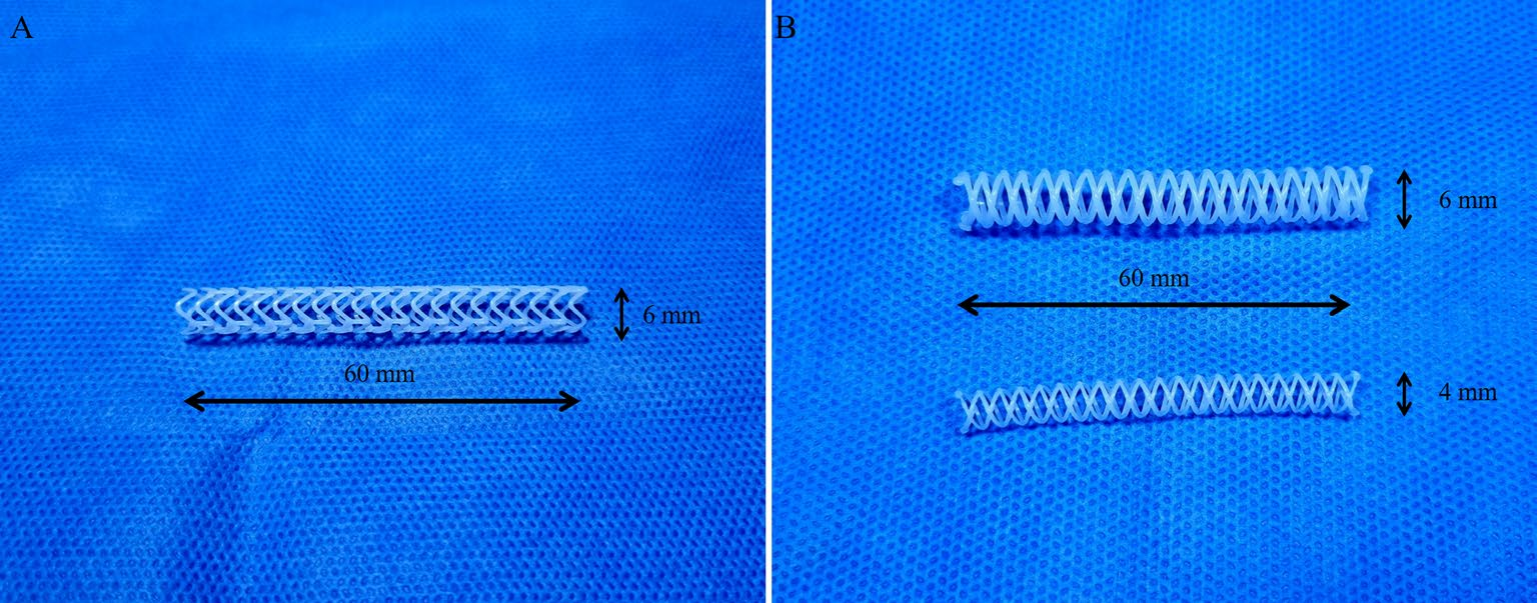
3D-printed polycaprolactone biliary stents. (**A**) Prototype stent. (**B**) Final version stent.

A rotating rod combined with a 3D printing system composed of a driving unit and extrusion unit was used to manufacture biodegradable stents^16^. The driving unit was operated by a G-code-based robot that consists of two heads moving translationally in the z-direction on the moving parts of x and y. The rotating rod used to print the stent was mounted on a rotating axis. The extrusion unit was composed of a heater and dispenser. The heater heated the material in the syringe to 250 °C. The molten material was extruded using a dispenser capable of providing pneumatic pressure from 0 to 700 kPa. The extrusion volume of the material could be modified using pneumatic pressure and nozzle size. The resolution of the 3D printing system is depending on printing temperature, extrusion pressure, and moving speed. It is possible to print the polymer from 150 μm to 1.5 mm. A rod was used as the substrate to print the tubular structure. Barium-loaded molten PCL was printed on a rotating rod to fabricate a stent with a strut of 500 µm. Our prototype PCL stent was 6 mm in diameter and 6 cm in length, and its shape was capable of loading into the delivery system used for percutaneous biliary intervention (Fig. 1A). However, the surgeon had a difficulty to insert prototype stent into the common bile duct (CBD) due to high flexibility of the stent. Therefore, we changed the design of the stent to make it easier to insert surgically. Considering the variation in CBD size depending on the pig, we made the final version PCL stent with a diameter of 4 mm or 6 mm and a length of 6 cm (Fig. 1B). The PDO (Resomer^®^ X 206 S; CAS 29223-92-5, Sigma-Aldrich, St. Louis, MO, USA) stents for the radial force comparison test were fabricated in the same manner.

### Stent radial force test

The radial force of the PDO and PCL stents during degradation in normal saline at 37 °C was examined. Radial force measurements of the two stents in each group (PDO and PCL) were performed after 5, 10, and 15 weeks. A compression test machine (Instron; Norwood, MA, USA) was used. The compressive velocity was 0.5 mm/s. The stent was fixed with a jig to prevent migration during measurement. Thereafter, the tubular-shaped probe compressed the middle of the stent in a cross-sectional direction to measure radial force.

### In vivo animal experiment

**Figure 2:**
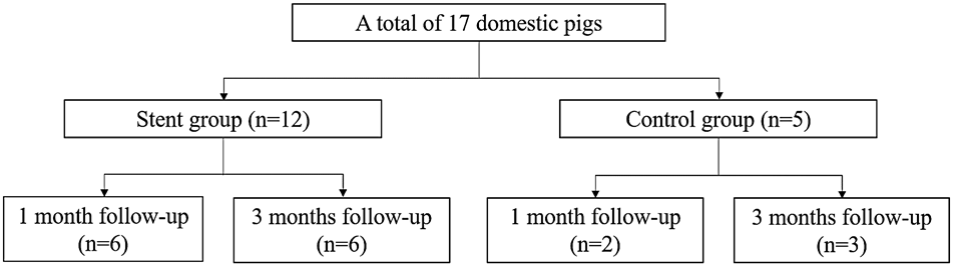
Flow diagram of animal allocation.

A total of 17 domestic male pigs (age, 3 months; weight, 26–30 kg) were divided into a stent group with 1-month follow-up (n = 6), stent group with a 3-month follow-up (n = 6), control group with a 1-month follow-up (n = 2), and control group with a 3-month follow-up (n = 3) (Fig. 2). 3 of the 6 stents used in stent group with a 3-month follow-up were prototype. All other stents were final version.

**Figure 3:**
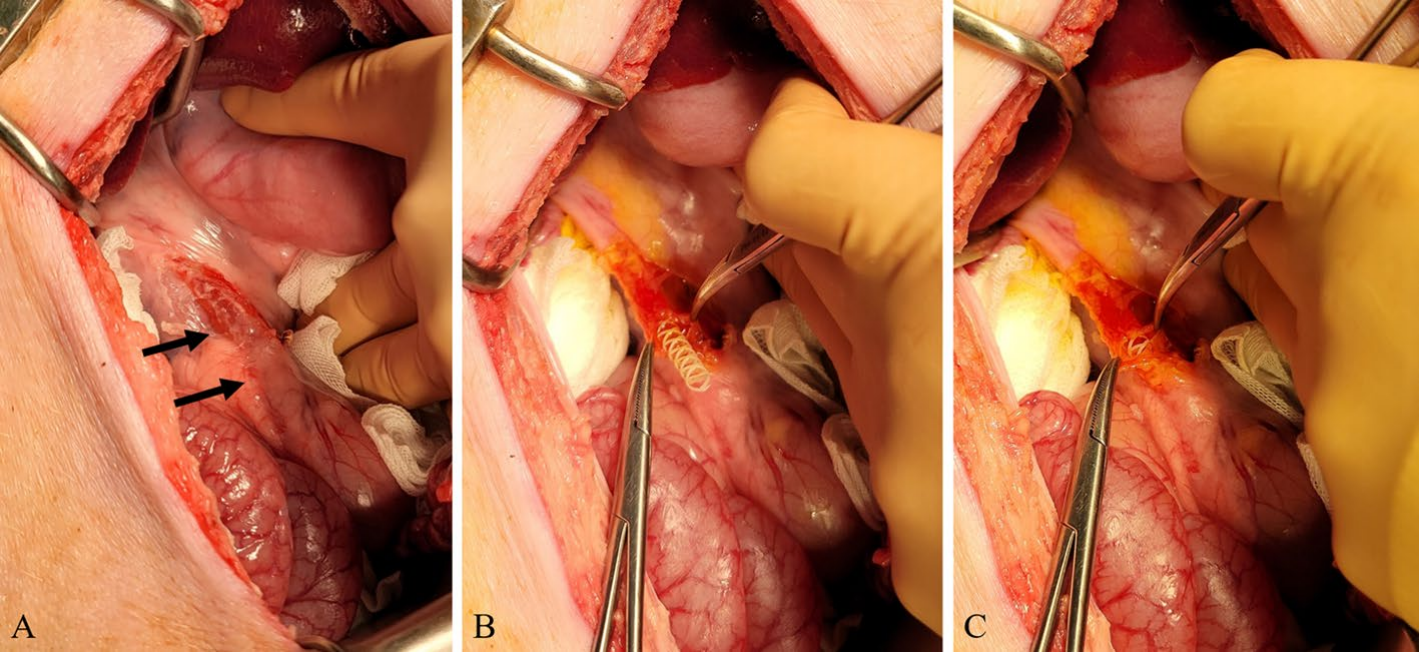
Stent placement in the common bile duct (CBD). (**A**) Identification and exposure of the CBD (arrows). (**B**,**C**) Transverse incision of the CBD and stent insertion.

All surgical procedures were performed by two experienced hepatobiliary surgeons (Y.C. and E.S.H.). Anesthesia was induced using intramuscular injection of tiletamine-zolazepam (2 mg/kg; Zoletil 50, Virbac S.A., Carros, France) and xylazine (2 mg/kg; Rompun, Bayer HealthCare, Leverkusen, Germany). Thereafter, endotracheal intubation was performed by a veterinarian and general anesthesia was achieved using 1.5–2% isoflurane and oxygen. After thorough shaving and skin preparation, a sterile drape was applied to the area. A midline upper laparotomy was performed and the CBD was identified and exposed. The CBD was opened transversely (Fig. 3). In the control group, the CBD incision was closed with interrupted sutures using monofilaments (polydioxanone, PDS II 5-0, Ethicone, Somerville, NJ, USA). In the stent group, a 4-mm or 6-mm biodegradable biliary stent was inserted into the CBD according to the bile duct diameter (Fig. 3). When the CBD length was less than 6 cm, the stent was cut with scissors to fit the CBD. CBD incision-site closure was performed in the same manner as in the control group. The laparotomy was closed with multifilament and monofilament sutures in the peritoneum, fascia, and subcutaneous layers (polyglactin, Vicryl 0, Ethicone, Somerville, NJ, USA), and skin (nylon, Ethilon 3-0, Ethicone, Somerville, NJ, USA).

Animals were evaluated daily. Postoperative analgesia was administered via intramuscular injection of meloxicam (Metacam, Boehringer Ingelheim, Burlington, ON, Canada) at 0.4 mL/10 kg for 3 days. All pigs also received an intramuscular injection of cefazolin (Cefozolin, Yuhan, Seoul, Korea) at 20 mg/kg, twice per day for 3 days to prevent infection. Pigs underwent 1-month and 3-month follow-up CT examinations to evaluate the status of biodegradable biliary stents and the presence of postoperative complications, such as bile duct dilatation and biloma formation.

Finally, the pigs were euthanized 1 or 3 months after surgical implantation with intravenous injection of potassium chloride (2 mmol/kg) prior to intramuscular injection of 2 mg/kg tiletamine-zolazepam (Zoletil 50, Virbac S.A., Carros, France) and 2 mg/kg xylazine (Rompun, Bayer HealthCare, Leverkusen, Germany) for sedation. The entire CBD was sharply extirpated and immersed in 10% buffered formalin for histopathological investigation.

### CT acquisition

CT examinations were performed using a commercially available, 64-channel CT scanner (Discovery CT 750 HD, GE Healthcare, Waukesha, WI, USA). Only non-contrast images were obtained to prevent unexpected adverse events in pigs following contrast injection. The CT parameters were as follows: detector configuration of 0.625 mm, tube voltage of 120 kVp, tube current of 150–200 mAs, and rotation time of 0.5 s. Axial images were reconstructed using a 2.5-mm slice thickness, and coronal multiplanar reconstruction (MPR) images were obtained using 3D imaging software.

### Histopathology

CBD specimens were fixed for at least 72 h in 10% buffered formalin, processed following standard histologic procedure, and embedded in paraffin blocks. Hematoxylin and eosin (H&E) and Masson’s trichrome staining were performed on 4-µm thick sections. On H&E staining, the severity of tissue lesions suggestive of an inflammatory response of the bile duct, including (a) epithelial hyperplasia, (b) mucinous gland hyperplasia, (c) lymphoplasmacytic infiltration, and (d) neutrophil infiltration, was graded on a scale of 0–3 (Table 1)^17^. The items (a), (b), and (c) indicate chronic inflammation, while (d) reflects acute inflammation. The sum of these four item scores was calculated as the total tissue inflammation score for each specimen. On Masson’s trichrome staining, the degree of fibrosis was evaluated by measuring submucosal fibrosis thickness. The mean of three measurements in the most severely fibrotic area was used as the representative value for each specimen. All histological examinations were performed by an experienced pathologist (J.K.) blinded to the specimen group.

**Table 1:** Grading system for the common bile duct tissue damage.

Grade	Epithelial hyperplasia	Mucinous gland hyperplasia	Lymphoplasmacytic infiltration	Neutrophil infiltration
0	Mucosa lined with a single layer of columnar epithelial cells	No significant mucinous gland hyperplasia	Spare lamina propria with occasional lymphocytes	None
1	Mild epithelial hyperplasia	Mild hyperplasia	Mild lymphoplasmacytic infiltration of lamina propria	Mild
2	Moderate epithelial hyperplasia	Moderate hyperplasia	Moderate lymphoplasmacytic infiltration of lamina propria	Moderate
3	Severe epithelial hyperplasia	Severe hyperplasia	Severe lymphoplasmacytic infiltration	Severe

### Statistical analysis

All results are reported as mean ± standard deviation. Differences in the tissue inflammation score and fibrosis thickness were compared using the Mann–Whitney *U* test. Statistical significance was set at P < 0.05. Statistical analyses were performed using MedCalc version 12 (MedCalc Software).

## Results

### Radial force test of 3D-printed biodegradable biliary stent

**Figure 4:**
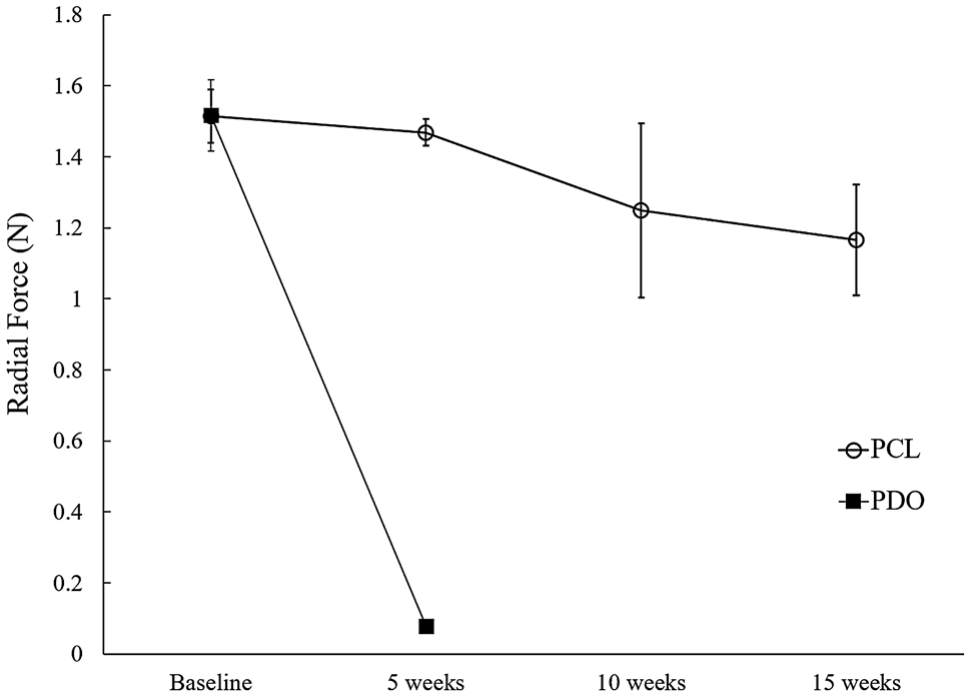
Change of the radial force in polycaprolactone (PCL) vs. polydioxanone (PDO) stents according to the degradation period.

The mean radial forces of PCL stents after 5, 10, and 15 weeks in 37 °C normal saline were 1.47 ± 0.04 N, 1.25 ± 0.25 N, and 1.17 ± 0.16 N, respectively (Fig. 4). The radial force of the untreated PCL stent (baseline) was 1.51 ± 0.08 N. Even after 15 weeks, the radial force of the PCL stent was more than 80% of the original radial force. In contrast, the PDO stent showed a rapid decrease in the radial force, with > 90% decrease in the initial value after 5 weeks (from 1.52 ± 0.10 N to 0.08 ± 0.001 N) (Fig. 4). It was difficult to measure the radial force of the PDO stents after 5 weeks because the structure was broken before measurement.

### Clinical and radiologic observation

All stents (n = 12) were successfully inserted into the CBD without major difficulties in surgical procedures. Partial resection and anastomosis of the CBD were easily performed in the control group (n = 5).

Among the five pigs in the control group, two died without an identifiable cause during the immediate postoperative period. Of the six pigs in the stent group with 1-month follow-up, one pig died on the first day of follow-up. Autopsy revealed a retained surgical gauze with a foreign body reaction in the abdominal cavity. In the remaining 14 pigs (six pigs in the stent group with a 3-month follow-up, five pigs in the stent group with a 1-month follow-up, two pigs in the control group with a 3-month follow-up, and one pig in the control group with a 1-month follow-up), no specific clinical symptoms or adverse events were observed during the follow-up period.

**Figure 5:**
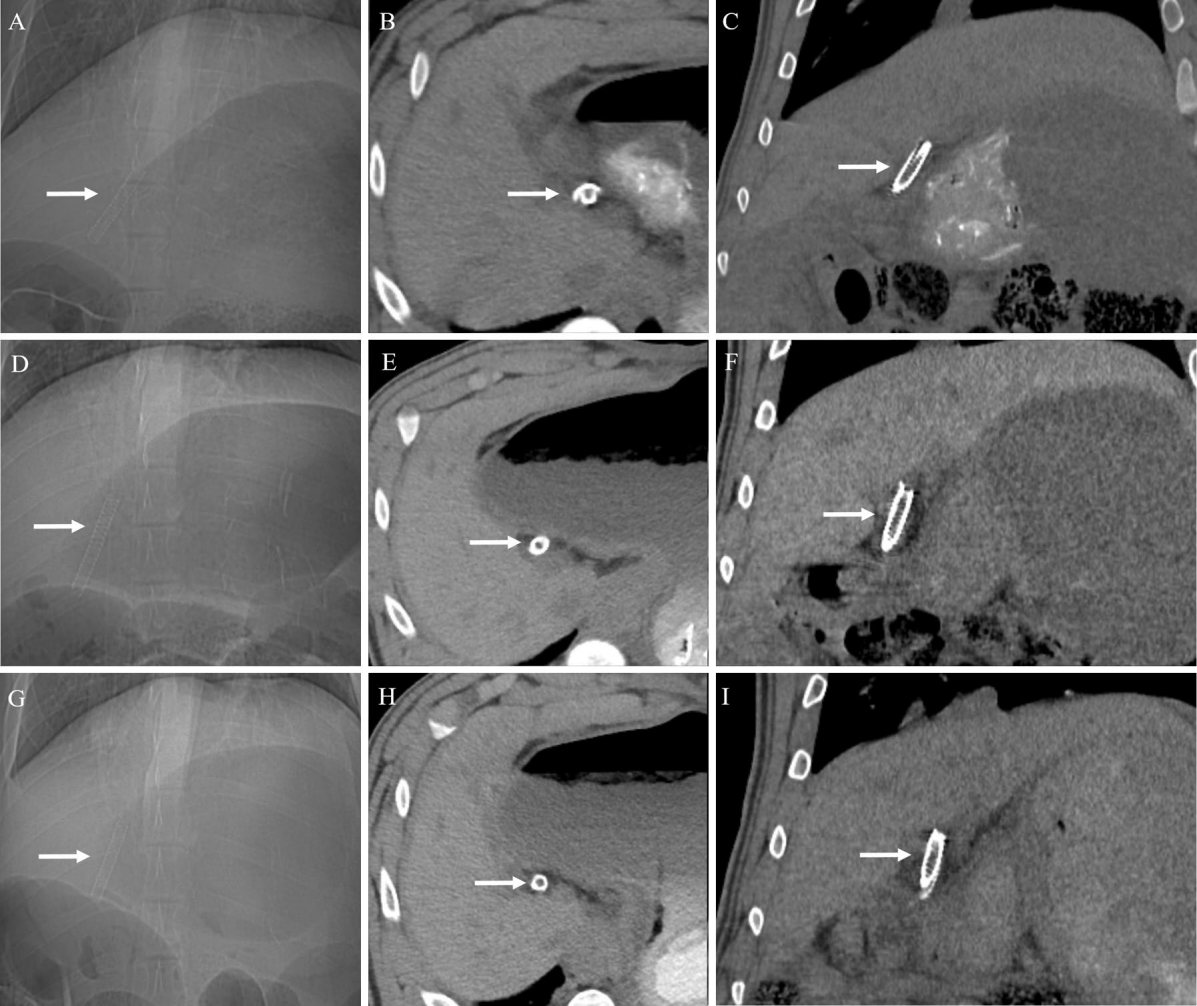
Representative images of 1-month (**A**–**C**), 2-month (**D**–**F**), and 3-month (**G**–**I**) follow-up CT scans to evaluate the status of biodegradable stents. The scout images (**A**,**D**,**G**), precontrast axial images (**B**,**E**,**H**), and coronal reconstruction images (**C**,**F**,**I**) show a radiopaque stent in the common bile duct without complications.

**Figure 6:**
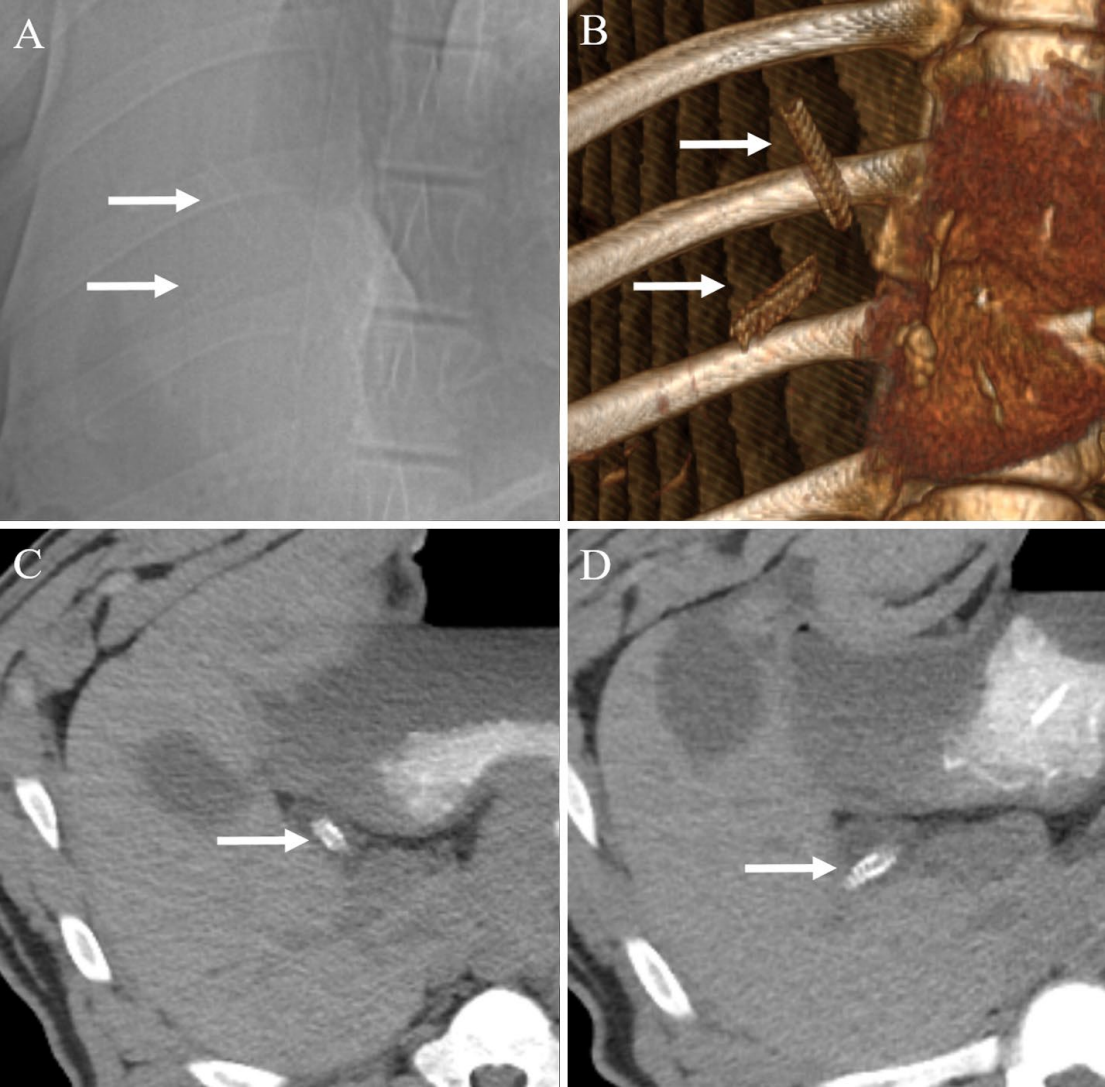
Two-month follow-up CT scan images showing stent fracture. (**A**) Scout image and (**B**) 3D-volume rendered image shows complete fracture of the stent (arrows). (**C**,**D**) Axial images also shows two fragments of fractured stent (arrows) within the common bile duct without any complications such as biliary obstruction or bile leakage.

On follow-up non-contrast CT examination, there was no radiologic evidence of bile duct obstruction or leakage in any pig (n = 14). Furthermore, the stents were clearly visualized as radiopaque material, which made it easy to confirm the position and condition of the stents (Fig. 5). Among the six pigs in the stent group with 3-month follow-up, in one pig, the stent disappeared on the 3-month follow-up CT. Of the five pigs in the stent group with 1-month follow-up, two pigs showed disappearance of stents on the 1-month follow-up CT. This might be due to distal migration of the stents following bile duct peristalsis. In addition, among the 11 pigs in the stent group, three pigs in the stent group with 3-month follow-up had a stent fracture on the 2-month follow-up CT (Fig. 6).

### Macroscopic evaluation of 3D-printed biodegradable biliary stent

**Figure 7:**
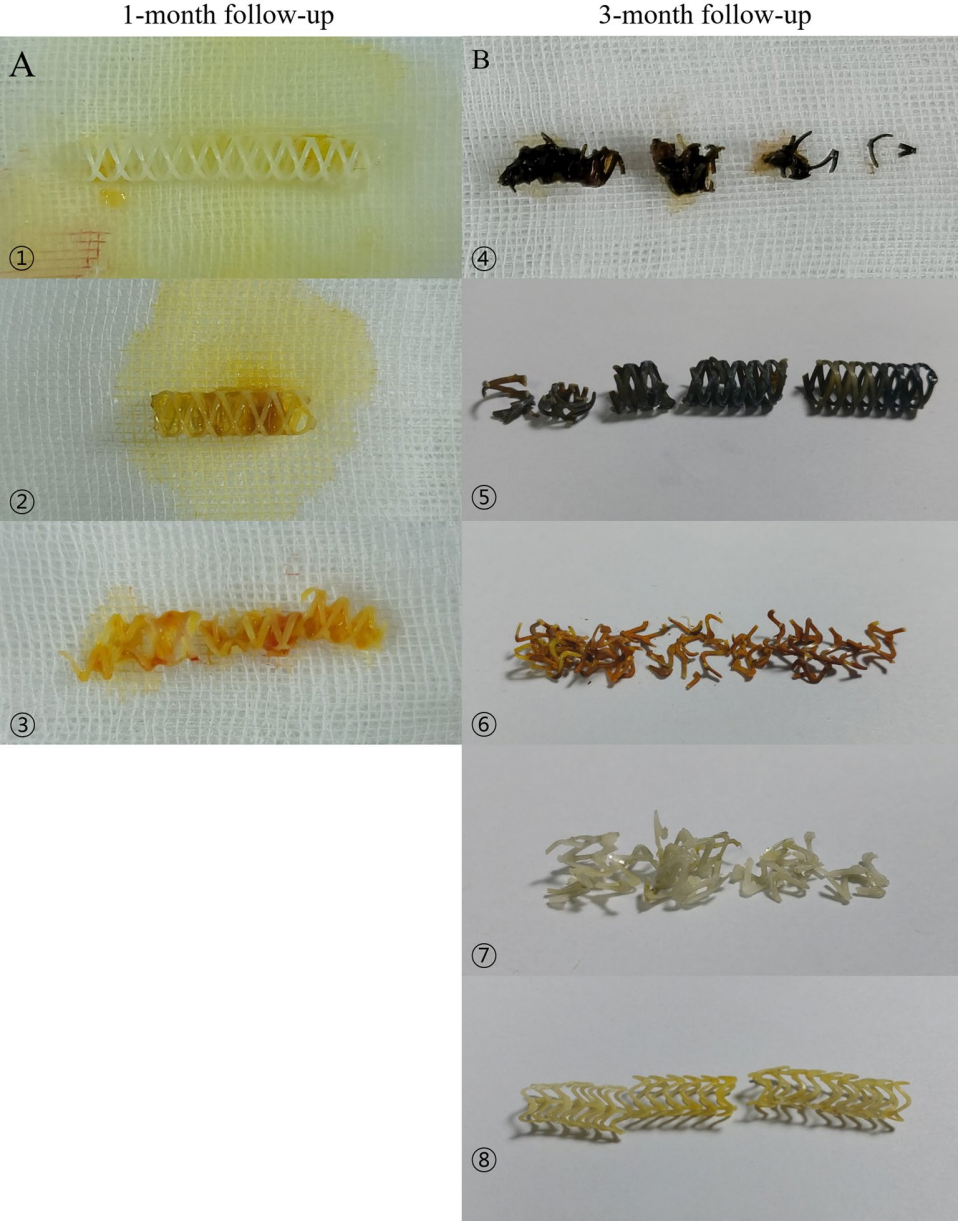
Gross morphology of polycaprolactone stents 1 month and 3 months after insertion. (**A**) Most stents maintain color and integration, 1 month after insertion. Specimen 3 was damaged by surgical instruments during common bile duct extraction. (**B**) At 3 months after stent insertion, most stents show discoloration and fragmentation. Specimens 6, 7, and 8 are prototype stents.

Macroscopic findings showed no definite change in color or integration of biodegradable biliary stents in the stent group at the 1-month follow-up (Fig. 7A). However, discoloration and fragmentation of biodegradable biliary stents were noted in the stent group at the 3-month follow-up (Fig. 7B).

### Histopathology

**Figure 8:**
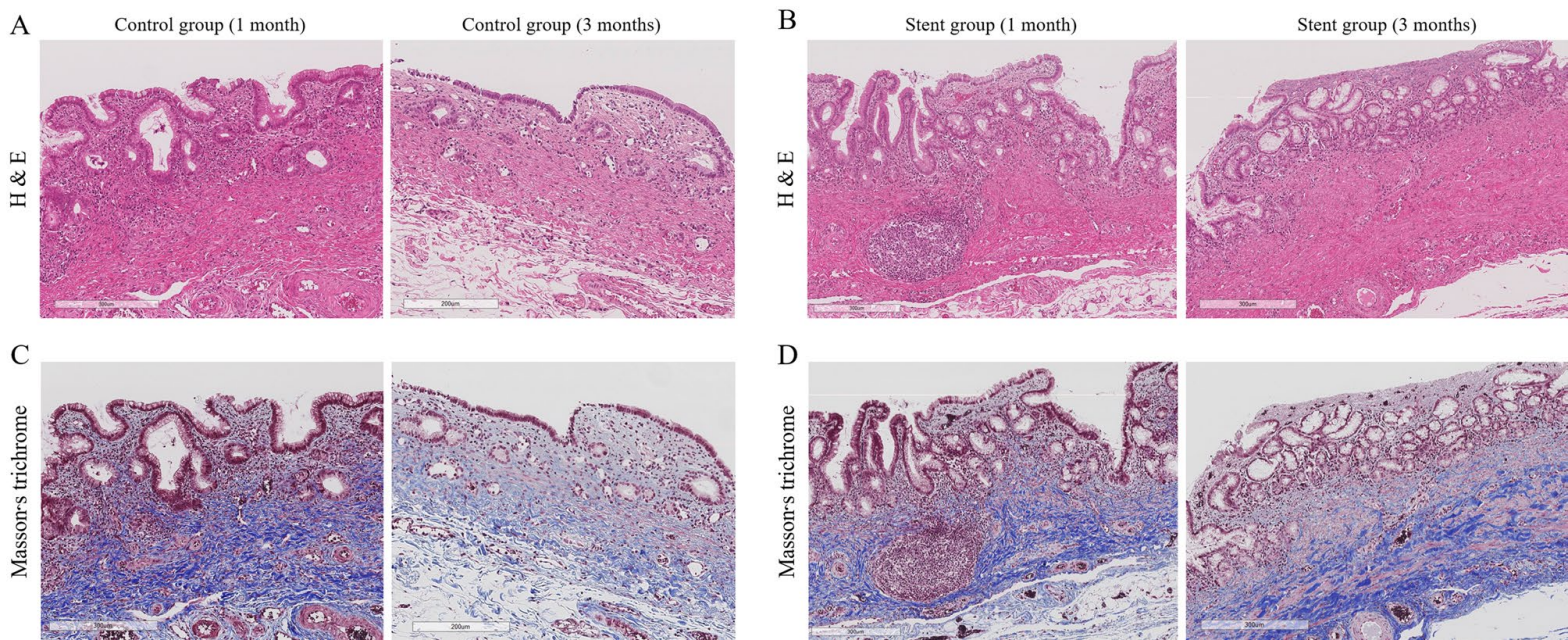
H&E and Masson’s trichrome staining of common bile ducts (CBD) from the control and stent groups. (**A**,**B**) H&E staining shows more mucinous gland hyperplasia and lymphoplasmacytic infiltration in the CBD of stent group than that of control group. (**C**,**D**) Masson’s trichrome staining of the CBD shows more prominent fibrosis (blue-colored staining of the collagen fibers) in the stent group than in the control group.

Because the tissue response could vary depending on the duration of stent placement, three pigs that lost their stents during the follow-up period were excluded from the histopathologic analysis. Therefore, histopathological examination was performed on CBD specimens obtained from 11 pigs (control group, n = 3; stent group, n = 8). The stent group showed significantly higher scores for mucinous gland hyperplasia, lymphoplasmacytic infiltration, and total tissue inflammation (P < 0.05) (Table 2; Fig. 8). There were no significant differences in epithelial hyperplasia or neutrophil infiltration between the stent and control groups. In addition, Masson’s trichrome staining showed thicker fibrosis in the stent group than in the control group (0.46 ± 0.12 mm vs. 0.21 ± 0.05 mm, P = 0.012) (Table 2; Fig. 8). Regarding the stent group, there was no difference in total tissue inflammation scores between the 1-month follow-up and 3-month follow-up groups (6.0 ± 2.65 vs. 5.4 ± 1.95; P = 0.763). In addition, there was no significant difference in fibrosis thickness between stent groups at the 1- and 3-month follow-up.

**Table 2:** Comparison of degree of tissue damage and fibrosis between stent and control groups.

	Stent group (n = 8)	Control group (n = 3)	P value
Epithelial hyperplasia	1.88 ± 0.83	1.33 ± 0.58	0.322
Mucinous gland hyperplasia	1.50 ± 0.53	0.33 ± 0.58	0.028
Lymphoplasmacytic infiltration	2.00 ± 0.76	0.33 ± 0.58	0.020
Neutrophil infiltration	0.25 ± 0.46	0.00 ± 0.00	0.361
Total tissue inflammation score	5.63 ± 2.07	2.00 ± 1.73	0.039
Thickness of fibrosis (mm)	0.46 ± 0.12	0.21 ± 0.05	0.012

Data are mean ± standard deviation.

## Discussion

Benign biliary strictures most commonly occur postoperatively, such as cholecystectomy and biliary anastomosis following LT. For the effective management of benign biliary strictures, repeated interventional procedures are necessary^18–20^. Although it is technically demanding and burdensome, balloon dilatation and progressive plastic stent insertion showed only 80% long-term durable stricture dilatation^21^. Biodegradable stents may overcome this disadvantage of conventional treatment by rendering repeated procedures for stent removal or unnecessary exchange. This study aimed to investigate the biocompatibility and safety of a novel 3D-printing biodegradable stent with PCL in a porcine model.

In our study, there were no biliary complications, such as obstruction or bile leakage, or adverse events during the 3-month follow-up period after stent placement in the CBD in a porcine model. This result suggests that our 3D printed biodegradable biliary stent made of PCL is biocompatible, at least in the porcine model, and is highly unlikely to cause problems in humans. This is similar to the results of previous studies showing good biocompatibility of biliary stents made using PDO during 8- or 20-week follow-up periods in a porcine model^17,22^. Regarding human application, Mauri et al.^10^ demonstrated the safety and effectiveness of the PDO stents for benign biliary strictures with an over 80% patency rate after monitoring 107 cases for two years.

We also demonstrated that the position and state of the biliary stent with barium could be easily evaluated on follow-up non-contrast CT. There was no difficulty in evaluating the position of the stent using only scout images. Visualization in imaging studies, such as plain radiography or CT, is one of the biggest issues in the use of biodegradable stents. Metallic stents are easily seen on plain radiography or CT because they are made of radiopaque materials. However, biodegradable materials cannot be visualized as X-rays penetrate the structure. In a previous study^22^, this problem was solved by indirectly visualizing the stent by attaching gold markers to either end of the stent. However, this method has limitations when evaluating the entire stent. Because our biodegradable stent was prepared by mixing barium powder directly in the molten PCL polymer, it was easy to view the entire stent on plain radiography or CT.

Regarding mechanical properties, in our study, the PDO stent demonstrated a rapid decrease in radial force, with a > 90% decrease from the initial value within 5 weeks. In contrast, the PCL stent maintained > 80% of the initial radial force even after 15 weeks. This is similar to a previous study^12^ in which PDO-dominant scaffolds lost more than 50% of their mechanical strength within 14 days, whereas PCL-dominant polymers maintained more than 90% of their initial strength after 56 days. According to several previous studies^23,24^, for the treatment of benign biliary strictures, the use of large-bore catheters maintained in place for over 6 months is necessary to achieve stricture remodeling. This finding suggests that an appropriate radial force should be applied to the stricture site for a long period to prevent stricture recurrence. Therefore, we believe that our biodegradable stents made with PCL could be more effective than PDO-based stents for the treatment of benign biliary strictures.

We found that stent migration occurred in 27.3% (3/11) of pigs, and 27.3% (3/11) of pigs exhibited stent fracture on 2-month follow-up CT. According to previous studies, stent migration occur in 16.2% of patients with fully-covered, self-expandable metal stents (CSEMS)^25^ and 1.9% of patients with PDO stents^10^ for the treatment of benign biliary stricture. Because balloon dilatation after stent placement was not performed in our study, the stent might not have been fixed well, resulting in a relatively high stent migration rate. In addition, stent migration can be prevented by changing the stent design through the addition of an anchoring flap^26^. Tomishima et al.^27^ reported that stent fracture occurred in 7.7% (2/26) after placement of CSEMS for benign biliary stricture. Although there have been no studies on the fracture rate of biodegradable biliary stents, in a previous human study using PDO stents^28^, 25% of patients developed mild cholangitis during stent indwelling. Siiki et al. reported that degradation and rupture of the stent may cause intermittent obstruction of bile flow, resulting in acute cholangitis^29^. However, blockage of the large bile duct with stent fragments or stone formation may be a minor complication, as most cases can be resolved with endoscopic procedures. In addition, the use of a stent tailored to the patient’s bile duct anatomy using 3D printing technology is expected to reduce the stent fracture rate. Although in our study, there were no complications related to stent fracture or fragmentation, such as bile duct obstruction or restenosis, further study is needed to develop a new PCL stent design with improved flexibility and fracture resistance.

In the gross specimens, our biodegradable biliary stents appeared fragmented at 3 months. However, considering that stents were well maintained in its original shape at 3 months follow-up CT, the process of taking the stents out of the CBD might have fragmented the fragile stents which were degraded for 3 months.

In terms of histopathology, although the stent group had significantly higher scores for mucinous gland hyperplasia and lymphoplasmacytic infiltration than those of the control group, the stent group’s scores also suggested mild-to-moderate inflammation. These results are consistent with those of a previous animal study using PDO biliary stents^17^, which showed mild-to-moderate inflammation during a 20-week follow-up period. In addition, fibrosis in the stent group was significantly thicker than that in the control group. According to another previous animal study using PDO biliary stents^22^, the degree of fibrosis in the control group was more marked than that in the stent group at the duct-to-duct biliary anastomosis site. However, in that previous study, the fibrosis thickness of the control group was 3.84 mm, which was significantly thicker than that of our study’s stent group (0.46 mm). Even the thickness of fibrosis in the stent group in our study was less than that of the control group (0.68 mm) in that study. Therefore, although there was a difference in fibrosis thickness between the control and stent groups in our study, the degree of fibrosis in the stent group was minimal. Furthermore, according to previous studies^29,30^, mild inflammation and fibrosis in the porcine bile duct that did not cause problems such as biliary obstruction or leakage were interpreted as acceptable in terms of safety and feasibility.

Our study had some limitations. First, because of the expense and resources involved in conducting large animal experiments, only 17 animals were studied. It would be necessary to perform larger studies to validate our findings. Second, we only evaluated the biocompatibility of PCL stents in an animal model, and the results of a human study might be different from those of our study. Third, we did not evaluate the effectiveness of PCL stents in a benign biliary stricture model. Further studies are required to clarify whether PCL stents can improve benign biliary strictures. Fourth, although PCL requires 3 years for complete removal in the body^12,31^ and stricture remodeling requires over 6 months after stent placement^2^, we only evaluated clinical and histopathologic outcomes after 3 months of follow-up. Further studies are required to confirm the long-term effects of PCL stent placement in the bile duct.

In conclusion, our novel 3D-printed PCL stent showed good biocompatibility after implantation in porcine bile ducts and has potential for use in the prevention of benign biliary strictures.

## Data Availability

The datasets generated during and/or analysed during the current study are available from the corresponding author on reasonable request.
